# 

**Published:** 1855-07

**Authors:** 


VOL. II.] JUNE & JULY, 1855. [NO. V.

### IOWA MEDICAL JOURNAL.

CONDUCTED BY THE FACULTY OF THE

MEDICAL DEPARTMENT OF THE IOWA UNIVERSITY.

PUBLISHED EVERY ALTERNATE MONTH

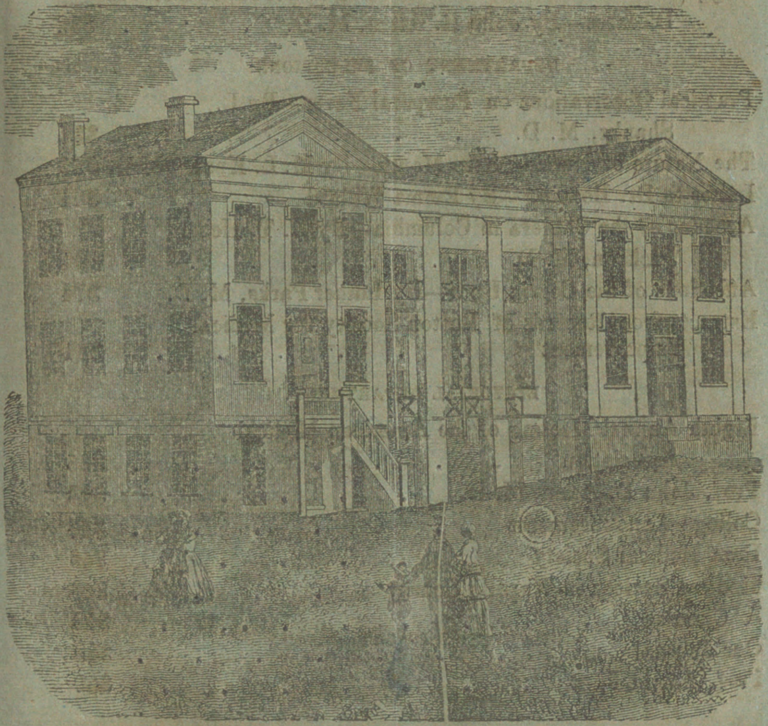

TERMS—TWO DOLLARS, IN ADVANCE.

KEOKUK, IOWA:

PRINTED AT THE WHIG BOOK AND JOB OFFICE.

Address all Communications, post-paid, to "Medical College, Box 109; Keokuk, Iowa."

